# Prevalence of Rotavirus Genotypes in Children Younger than 5 Years of Age before the Introduction of a Universal Rotavirus Vaccination Program: Report of Rotavirus Surveillance in Turkey

**DOI:** 10.1371/journal.pone.0113674

**Published:** 2014-12-01

**Authors:** Riza Durmaz, Atila Taner Kalaycioglu, Sumeyra Acar, Zekiye Bakkaloglu, Alper Karagoz, Gulay Korukluoglu, Mustafa Ertek, Mehmet Ali Torunoglu, the Turkish Rotavirus Surveillance Network

**Affiliations:** 1 Molecular Microbiology Research and Applied Laboratory, Public Health Agency of Turkey, Ankara, Turkey; 2 Department of Medical Microbiology, Faculty of Medicine Yıldırım Beyazıt University, Ankara, Turkey; 3 Virology Reference Central Laboratory, Public Health Agency of Turkey, Ankara, Turkey; Centers for Disease Control and Prevention, United States of America

## Abstract

**Background:**

Group A rotaviruses are the most common causative agent of acute gastroenteritis among children less than 5 years of age throughout the world. This sentinel surveillance study was aimed to obtain baseline data on the rotavirus G and P genotypes across Turkey before the introduction of a universal rotavirus vaccination program.

**Methods:**

Rotavirus antigen-positive samples were collected from 2102 children less than 5 years of age who attended hospitals participating in the Turkish Rotavirus Surveillance Network. Rotavirus antigen was detected in the laboratories of participating hospitals by commercial serological tests such as latex agglutination, immunochromatographic test or enzyme immunoassay. Rotavirus G and P genotypes were determined by reverse transcription polymerase chain reaction (RT-PCR) using consensus primers detecting the VP7 and VP4 genes, followed by semi-nested type-specific multiplex PCR.

**Results:**

RT-PCR found rotavirus RNA in 1644 (78.2%) of the samples tested. The highest rate of rotavirus positivity (38.7%) was observed among children in the 13 to 24 month age group, followed by children in the age group of 25 to 36 months (28.3%). A total of eight different G types, six different P types, and 42 different G–P combinations were obtained. Four common G types (G1, G2, G3, and G9) and two common P types (P[8] and P[4]) accounted for 95.1% and 98.8% of the strains, respectively. G9P[8] was the most common G/P combination found in 40.5% of the strains followed by G1P[8] (21.6%), G2P[8] (9.3%), G2P[4] (6.5%), G3P[8] (3.5%), and finally, G4P[8] (3.4%). These six common genotypes included 83.7% of the strains tested in this study. The rate of uncommon genotypes was 14%.

**Conclusion:**

The majority of the strains analyzed belonged to the G1–G4 and G9 genotypes, suggesting high coverage of current rotavirus vaccines. This study also demonstrates a dramatic increase in G9 genotype across the country.

## Introduction

Rotaviruses are the most important causative agents of severe gastroenteritis in infants and young children worldwide and are responsible for 453,000 deaths in 2008 [1]. More than one-third of deaths attributable to diarrhea and 5% of all deaths in children less than 5 years of age are due to rotavirus infections [1]. They are responsible for 25% to 50% of all hospitalizations for diarrhea in children in both developed and developing countries [2]. Although mortality as a result of rotavirus infection is low in countries with good health care facilities, an 85% mortality rate has been reported in South Asia and sub-Saharan Africa [3].

Rotaviruses belong to the *Reoviridae* family and have 11 segments of double-stranded RNA surrounded by a triple-layered capsid containing a core, inner, and outer capsid. Based on the antigenic and genetic features of the inner capsid protein VP6, rotaviruses are categorized into seven major groups (A–G). Most human rotaviruses belong to group A. The glycoprotein VP4 and the protease-sensitive VP7, which are structural proteins on the outer capsid, define the virus G and P genotypes, respectively [4], [5]. The VP7 and VP4 proteins are also very important for host specificity, virulence and neutralizing antibody response [4], [6]. Due to the segmented nature of the viral genome, reassortment events are possible between two strains infecting the same host resulting in the production of novel P and G rotavirus genotypes [6], [7]. To date, at least 27 G-types, 35 P-types and 42 different G–P type combinations have been detected [4], [8]. Although the prevalence of the genotypes shows variation from year to year and from one geographic area to another, only a few G–P combinations including G1P [8], G2P[4], G3P[8], G4P[8], and G9P[8] are prevalent in humans around the world [9], [10]. Uncommon genotypes, such as G12P [8], G12P[6], G2P[8], G4P[6], and G3P[6], have been reported with lower rates in different countries [6], [11], [12].

Because the VP4 and VP7 surface proteins elicit neutralizing antibodies *in vivo*, they are the target molecules for the development of vaccines. Currently, there are two oral live attenuated rotavirus vaccines: Rotarix (GlaxoSmithKline Biologicals, Rixensart, Belgium) and RotaTeq (Merck & Co., Inc., Whitehouse Station, NJ, USA). Rotarix is a monovalent vaccine derived from the human rotavirus strain G1P[8] [13]. RotaTeq is a pentavalent human-bovine vaccine consisting of G1, G2, G3, G4 and P[8] genotypes, which are the most common human types [14]. Both vaccines are available in Turkey; however, they have not been introduced in national vaccination programs. Before the introduction of universal rotavirus vaccines, it is essential to document the circulating genotypes. This will enable monitoring of the effects of vaccines on the diversity of rotavirus genotypes and identify the emergence of genotypes that escape vaccine-induced immunity [15].

In Turkey, acute gastroenteritis is a major public health concern affecting more than 352,000 children less than 5 years old annually (http://tsim.saglik.gov.tr/tsim.htm). Rotavirus positivity in children with gastroenteritis has been reported to be among 21%−43.6% [16]–[22]. Several studies carried out in local areas showed that the majority of rotavirus strains were classified in G1–G4 genotypes between 2000 and 2010 in Turkey [18], [20], [21], [23]. Two recent studies performed in the Ankara province of Turkey highlighted the increased prevalence of the G9P[8] genotype [17], [19].

Given the marked fluctuation in circulating rotavirus genotypes in different study periods and populations [9], [11], [12], [24]–[26], the continued surveillance programs pre- and post-vaccination era can provide useful data for monitoring the changes in rotavirus disease burden and circulating genotypes over time and evaluate the efficacy of available vaccines. The aim of this study was to detect the prevalence of G and P genotype rotavirus strains collected in a two-year period of the sentinel surveillance program carried out in 23 Turkish provinces and to confirm baseline data regarding circulating genotypes before the introduction of a national rotavirus vaccination program.

## Materials and Methods

### Study population

This sentinel surveillance study was conducted in 35 hospitals in 23 provinces around the country from August 2012 to July 2014. The locations of these provinces corresponding to the seven geographical regions are as follows: Istanbul and Bursa provinces are in the Marmara region; Izmir and Afyon in the Aegean Region; Ankara, Eskisehir, Kayseri, and Konya in Central Anatolia; Antalya, Mersin, Isparta, and Adana in the Mediterranean region; Gaziantep, Sanlıurfa, and Diyarbakir in South-East Anatolia, Erzurum, Malatya, and Van in East Anatolia; Rize, Trabzon, Tokat, Samsun, and Karabuk in the Black Sea region. All participating hospitals collected fecal samples from the children less than 5 years of age who were admitted for treatment of acute gastroenteritis. Stool samples were collected in the first 48 hours of hospitalization and tested for the presence of rotavirus antigen in laboratories of participating hospitals by commercial serological tests such as latex agglutination (Rotalex, Orion Diagnostica), immunochromatographic test (Rota Uni-Strip, Coris BioConcept; Vikia Rota-Adeno, BioMerieux) or enzyme immunoassay (Ridascreen rotavirus, R-Biopharm, Darmstadt, Germany). Antigen-positive stool samples were then transferred to the Public Health Agency of Turkey (PHAT) for genotyping. All available information such as age and sex of patients, date of sample collection, symptoms, geographical location and participating hospital were recorded, and the patient’s information forms were sent to PHAT together with the fecal samples.

### Ethics Statement

This study was approved by the ethics committee of the Ministry of Health, Dr. Abdurrahman Yurtaslan Oncology Training and Research Hospital, Ankara/Turkey (Protocol code: 2012-8/37). The verbal consent of the mother or the guardian of the child enrolled in this study was obtained prior to the sample collection. Because collection of fecal samples from children with suspected rotavirus infection being admitted to the hospital was a routine process for rotavirus diagnosis, verbal consent was approved by the ethics committees.

### Genotyping by semi-nested multiplex RT-PCR

#### RNA extraction

A 10% (w/v) suspension of antigen-positive stool samples was prepared in phosphate-buffered saline (PBS). The fecal suspension was vortexed and centrifuged at 3000×g for 15 min. The supernatant was then used for RNA extraction by an EZ1 virus Mini Kit *(Qiagen GmbH, Hilden, Germany)* in accordance with the manufacturer’s instructions.

#### G and P Genotyping

All antigen-positive samples were subjected to RT-PCR with consensus primers VP7-forward/VP7-reverse and VP4-forward/VP4-reverse to amplify the VP7 and VP4 genes, respectively [6], [27]. For amplification of the VP7 gene, 5 µL of extracted RNA was reverse-transcribed and amplified using the Superscript one-step RT- PCR kit (Invitrogen) in the presence of 20 pmol of each primer, in particular, the VP7-forward and VP7-reverse primers described by Iturriza-Gómara et al. [6]. The thermal-cycling was performed as follows: denaturation of dsRNA at 95°C for 5 min, reverse transcription at 45°C for 45 min, and then amplification of cDNA following the cycling parameters described by Iturriza-Gómara et al. [6]. For amplification of the VP4 gene, first cDNA was synthesized with random-hexamer primer using the first strand cDNA synthesis kit (Thermo Scientific, CA, USA). Then, the cDNA was amplified using 20 pmol of the VP4-forward/VP4-reverse primers described by Simmonds et al. [27] in the PCR master mix (Thermo Scientific, CA, USA). The amplification conditions were as follows: an initial denaturation at 95°C for 3 min, followed by 35 cycles at 95°C for 45 s, 54°C for 45 s, and 72°C for 1 min with a final extension step at 72°C for 10 min. Semi-nested type-specific multiplex PCR was used to identify P and G genotypes with the primers listed in Table 1. G typing was performed using 2 µL of the first-round PCR product, 20 pmol of each of specific primers targeted to G1, G2, G3, G4, G8, G9, and G10 and a VP7-R consensus primer in PCR master mix (Thermo Scientific, CA, USA) following the cycling conditions described by Iturriza-Gómara et al. [6]. P typing was performed using 2 µL of the first-round PCR product along with specific P[4] (10 pmol), P[6] (5 pmol), P[8] (15 pmol), P[9] (5 pmol), P[10] (5 pmol), and P[11] (5 pmol) primers with a VP4-F consensus primer (10 pmol). Thermal-cycling was performed including an initial denaturation at 95°C for 3 min followed by 35 cycles at 95°C for 45 s, 45°C for 45 s and 72°C for 1 min with a final extension step at 72°C for 10 min. The amplification product was electrophoresed through a 2% agarose gel, and genotypes were determined by the sizes of the amplicons. Sequences of the primers used and the amplicon sizes of each genotype are shown in Table 1.

**Table 1:** G and P consensus and type-specific primers.

Primers	Sequences (5′–3′)	Amplicon sizes
**G-typing (a)**		
1st round (Consensus)		
VP7-F	ATGTATGGTATTGAATATACCAC	881 (c)
VP7-R	AACTTGCCACCATTTTTTCC	
2nd round (type-specific)		
G1	CAAGTACTCAAATCAATGATGG	618 (c)
G2	CAATGATATTAACACATTTTCTGTG	521 (c)
G3	ACGAACTCAACACGAGAGG	682 (c)
G4	CGTTTCTGGTGAGGAGTTG	452 (c)
G8	GTCACACCATTTGTAAATTCG	754 (c)
G9	CTTGATGTGACTAYAAATAC	179 (c)
G10	ATGTCAGACTACARATACTGG	266 (c)
VP7-R	AACTTGCCACCATTTTTTCC	
**P-typing**		
1st round (Consensus) (b)		
VP4-F	TATGCTCCAGTNAATTGG	663 (d)
VP4R	ATTGCATTTCTTTCCATAATG	
2nd round (type-specific) (a)		
P[4]	CTATTGTTAGAGGTTAGAGTC	362 (e)
P[6]	TGTTGATTAGTTGGATTCAA	146 (e)
P[8]	TCTACTGGRTTRACNTGC	224 (e)
P[9]	TGAGACATGCAATTGGAC	270 (e)
P[10]	ATCATAGTTAGTAGTCGG	462 (e)
P[11]	GTAAACATCCAGAATGTG	191(e)
VP4-F (b)	TATGCTCCAGTNAATTGG	

a,b Primers were obtained from references 6 and 27. ^c,d^Amplicon sizes were obtained from references 6 and 27. ^e^Amplicon sizes were estimated using the nucleotide positions of forward and reverse primers.

### Sequencing

Sequencing was performed on the samples that produced amplicons in the first-round PCR with the consensus primers, but no genotype-specific products were obtained in the second round of the semi-nested multiplex PCR. After purification of the first-round PCR products with Agencourt AMpure (Beckman Coulter Company, Massachusetts, USA), the sequencing was performed using consensus primers for the VP7 and VP4 genes. Each sequence reaction consisted of 5 pmol of primer, 3.5–5 µL of purified amplicon, and 4 µL of dye terminator cycle sequencing quick start kit (Beckman Coulter, Massachusetts, USA). The sequencing reaction was performed as follows: initial denaturation at 94°C for 3 min, followed by 30 cycles of denaturation at 96°C for 20 s, annealing at 55°C for 20 s, and elongation at 60°C for 4 min. The sequencing products were purified with a dye-terminator removal kit (Agencourt CleanSEQ, Beckman Coulter Company, Massachusetts, USA) and the sequence data were collected from a Beckman Coulter CEQ 8000 genetic analysis and sequencing system. The VP4 and VP7 sequence results were compared with the VP4 and VP7 sequencing data available in the GenBank database (www.ncbi.nlm.gov/genbank).

### Statistical analysis

Statistical analysis was performed using the statistical program SPSS version 16.0. Differences in proportions of rotavirus positivity in different age groups, gender, and geographic regions were tested using the chi-square (χ^2^) test. A p-value of <0.05 was considered statistically significant.

## Results

### Characteristics of rotavirus positive samples

**Figure 1:**
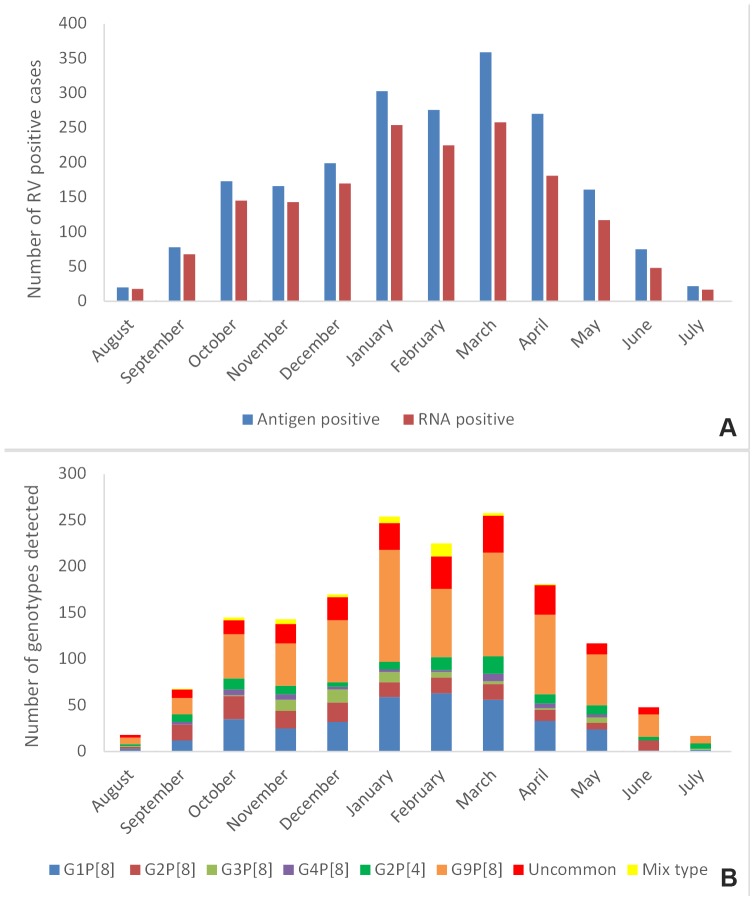
Seasonal distribution of rotavirus-positive samples (A) and G–P genotype combinations detected (B).

**Figure 2:**
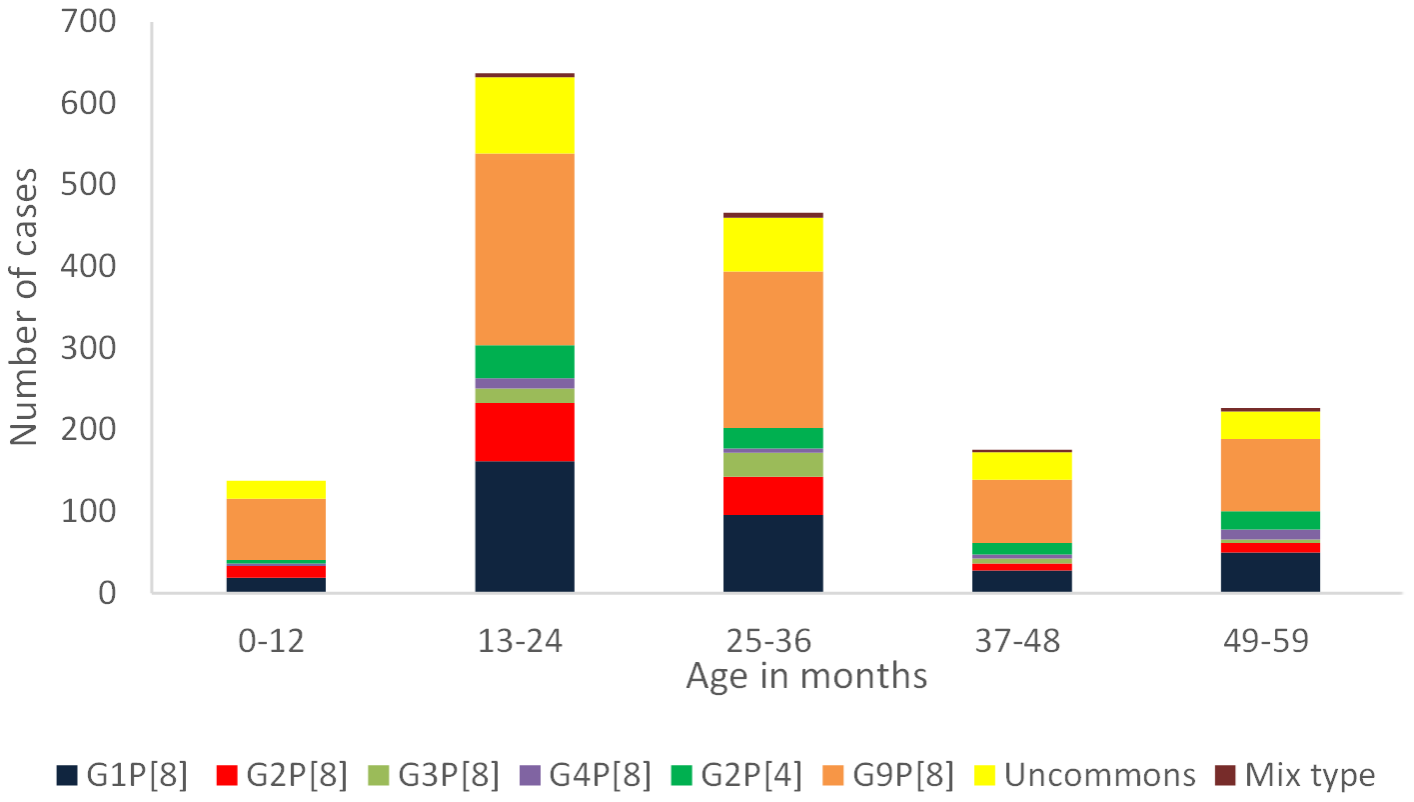
The total number of rotavirus RNA-positive samples and distribution of major genotypes in different age groups.

**Figure 3:**
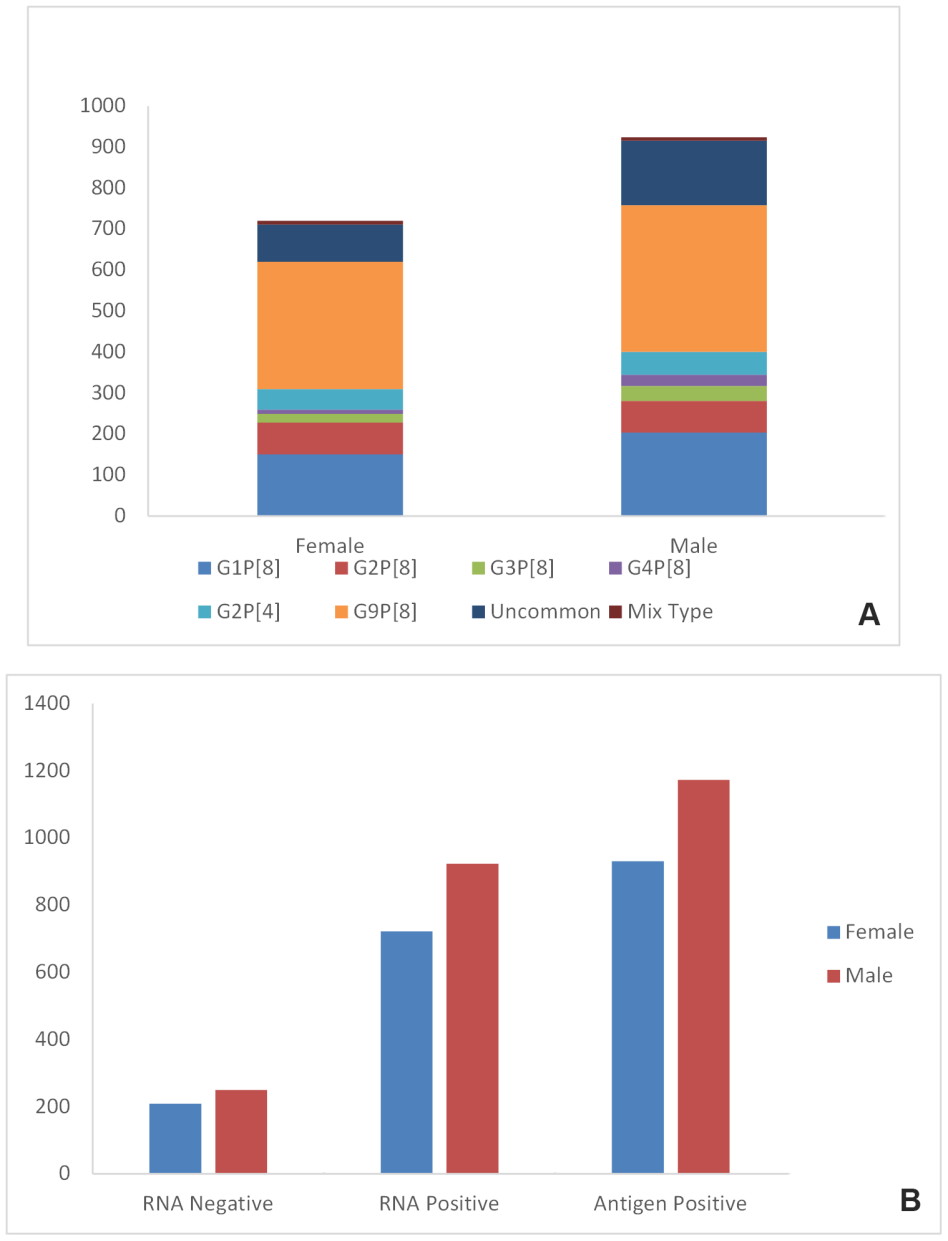
Rotavirus positivity (A) and genotype distribution (B) between females and males.

All antigen-positive stool samples (n = 2102) were obtained from children less than 5 years of age experiencing severe acute gastroenteritis throughout the year with a peak from September to the end of May. The highest sampling rate was 17.1% (359/2102) in March, followed by 14.4% (303/2102) in January, and 13.1% (276/2102) in February. The number of rotavirus antigen-positive cases varied from 1% to 9.5% in the remaining nine months (Figure 1A). Rotavirus RNA was detected in 1644 (78.2%) of the samples tested by RT-PCR, with the remaining 458 (21.8%) samples yielding negative results by both consensus RT-PCR and commercial real-time RT-PCR. Of the rotavirus RNA positive samples, 396 (24.1%) were collected from South-East Anatolia, 394 (24%) from Central Anatolia, 318 (19%) from the Marmara region, 194 (12%) from the Black Sea region, 160 (10%) from the Aegean region, 97 (6%) from the Mediterranean region, and 85 from East Anatolia (Table 2). The age of rotavirus RNA positive patients ranged from 15 days to 59 months with 138 children (8.4%) in the age group of 0 to 12 months, 637 (38.7%) in the age group of 13 to 24 months, 466 (28.3%) in the age group of 25 to 36 months, 176 (10.7%) in the age group of 37 to 48 months, and 227 (13.8%) in the age group of 49 to 59 months (Figure 2). The prevalence of rotavirus infection was significantly higher in the age group of 13 to 24 months than the other age groups (p<0.05). Of the 1644 children, 721 (43.9%) were female and the remaining 923 (56.1%) were male (Figure 3A). Rotavirus positivity did not differ significantly between females and males (p>0.05).

**Table 2:** Geographical distribution of 42 different rotavirus G and P genotype combinations in Turkey from August 2012 to July 2014.

	CentralAnatolia	EastAnatolia	South-EastAnatolia		Black Sea	Aegean	Marmara	Mediterranean	Total (%)
**Common** **genotypes**									
G1P[8]	59	32	98		55	25	66	20	355 (21.6)
G2P[8]	14	1	78		7	16	27	10	153 (9.3)
G3P[8]	18	0	24		3	1	9	2	57 (3.5)
G4P[8]	6	6	2		2	6	17	0	39 (3.4)
G2P[4]	17	4	44		23	3	8	8	107 (6.5)
G9P[8]	213	19	103		70	79	144	38	666 (40.5)
**Subtotal**	**327**	**62**	**349**		**160**	**130**	**271**	**78**	**1377 (83.7)**
**Uncommon** **genotypes**									
G10P[8]	0		0	2	1	0	2	0	5 (0.30)
G12P[11]	0		1	1	0	0	0	0	2 (0.12)
G12P[6]	0		2	0	0	0	0	0	2 (0.12)
G12P[8]	3		3	0	1	0	0	0	7 (0.42)
G1P[4]	9		9	7	8	5	13	4	55 (3.34)
G3P[4]	3		0	1	1	2	2	0	9 (0.54)
G4P[4]	0		0	0	1	1	2	0	4 (0.24)
G4P[9]	0		0	0	0	0	2	0	2 (0.12)
G8P[4]	1		0	0	1	0	0	0	2 (0.12)
G8P[8]	1		0	7	0	1	0	1	10 (0.60)
G9P[10]	1		0	0	0	0	0	0	1 (0.06)
G9P[4]	34		3	19	9	21	19	13	118 (7.2)
G9P[6]	0		1	3	0	0	0	0	4 (0.24)
G12P[N]	0		1	0	0	0	0	0	1 (0.06)
G9P[N]	0		0	0	1	0	1	0	2 (0.12)
G8P[N]	0		0	0	1	0	0	0	1(0.06)
GNP[8]	0		0	1	0	0	0	0	1 (0.06)
GNP[4]	0		0	0	0	0	1	0	1 (0.06)
G9P[9]	0		0	0	1	0	0	0	1 (0.06)
G1P[9]	0		0	0	2	0	1	0	3 (0.18)
**Subtotal**	52		20	41	27	30	43	18	231 (14)
**Mix** **genotypes**									
G10P[4]P[8]	1		0	0	0	0	0	0	1 (0.06)
G12P[6]P[8]	0		1	0	0	0	0	0	1 (0.06)
G1G2P[4]P[8]	0		0	0	1	0	0	0	1 (0.06)
G1G9P[4]	0		0	0	1	0	0	0	1 (0.06)
G1G9P[8]	1		1	0	0	0	1	0	3 (0.18)
G1P[4]P[8]	1		0	2	2	0	0	0	5 (0.30)
G1P[6]P[8]	0		1	0	0	0	0	0	1 (0.06)
G2G9P[8]	1		0	1	2	0	0	1	5 (0.30)
G2P[4]P[8]	2		0	1	0	0	0	0	3 (0.18)
G2G3P[4]	1		0	0	0	0	0	0	1 (0.06)
G3G9P[8]	1		0	0	0	0	2	0	3 (0.18)
G3P[4]P[8]	2		0	0	0	0	0	0	2 (0.12)
G4P[4]P[8]	1		0	0	0	0	0	0	1 (0.06)
G9G1P[8]	1		0	0	0	0	0	0	1 (0.06)
G1G3P[4]	0		0	0	0	0	1	0	1 (0.06)
G9P[4]P[8]	3		0	2	1	0	0	0	6 (0.36)
**Subtotal**	**15**		**3**	**6**	**7**	**0**	**4**	**1**	**36 (2.18)**
**TOTAL (%)**	394(24%)		85(5%)	396(24%)	194(12%)	160(10%)	318(19%)	97(6%)	1644

N, non-typeable G and/or P genotype; Mixed, presence of multiple genotypes in the same stools.

### Prevalence of rotavirus genotypes

Among the 1644 rotavirus PCR positive samples, six were partially typed. In four samples, the G genotype was defined but the P type remained negative, and in two samples, only the P type was determined. Both G and P genotypes were identified in a total of 1638 samples. A total of eight different G types, six different P types, and 42 different G–P combinations were observed. Among the G genotypes, G9 was the most frequently detected (n = 800, 48.7%), followed by G1 (n = 425, 25.9%), G2 (n = 267, 16.2%), G3 (n = 71, 4.3%), G4 (n = 45, 2.7%), G8 (n = 15, 0.9%), G12 (n = 13, 0.8%), and G10 (n = 6, 0.4%). G12 was detected for the first time in Turkey. The predominant P genotype was P[8] (n = 1305, 79.4%), followed by P[4] (n = 320, 19.5%), P[9] (n = 7, 0.43%), P [6] (n = 5, 0.30%), P[11] (n = 2, 0.12%), and P[10] (n = 1, 0.06%). Four common G types (G1, G2, G3, and G9) and two common P types (P[8], P[4]) accounted for 95.1% and 98.8% of the strains, respectively.

The most common G and P combinations in the samples tested were G9P[8] (n = 666, 40.5%), followed by G1P[8] (n = 355, 21.6%), G2P[8] (n = 153, 9.3%), G2P[4] (n = 107, 6.5%), G3P[8] (n = 57, 3.5%), and G4P[8] (n = 39, 3.4%). These six common genotypes included 1377 (83.7%) of the samples tested in this study. The remaining 231 samples (14.1%) belonged to one of the 20 uncommon combinations such as G9P[4] (n = 118, 7.2%), G1P[4] (n = 55, 3.3%), G8P[8] (n = 10, 0.6%), G12P[8] (n = 7, 0.4%), G3P[4] (n = 9, 0.5%), or G10P[8] (n = 5, 0.3%). A total of 16 different mixed genotypes, such as G9P[4]P[8] (n = 6, 0.4%), G1P[4]P[8] (n = 5, 0.3), and G2G9P[8] (n = 5, 0.3) were identified in the 36 samples (2.2%) (Table 2).

### Distribution of the common genotypes by age, sex, and month

There were no genotype restrictions in a specific age group, sex or month. We also did not find any large variations in the proportion of G and P genotypes detected in different months. G9P[8], G1P[8], and G2P[8] were three common genotypes detected in all months (Figure 1B). The proportion of each genotype identified for each age group was almost similar. For instance, G9P[8] and G1P[8] were the most and second-most common genotypes in all age groups, respectively (Figure 2). All common genotypes were detected in both females and males with similar frequencies. The proportion of G9P[8], G1P[8], G2P[8], and G2P[4] was 39%, 22%, 8%, and 6%, respectively, in males, whereas these values were 43%, 21%, 11%, and 7%, respectively, in females (Figure 3B).

### Distribution of common genotypes between geographic regions

**Figure 4:**
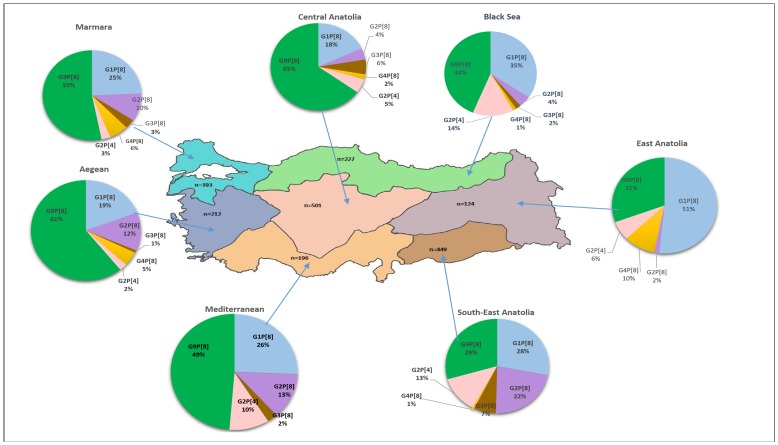
Distribution of common G and P genotype combinations among seven geographic regions of Turkey. The “n” indicates the total number of antigen-positive samples collected in each region.

Given geographic regions of Turkey have different climates and socio-economic conditions, we evaluated the regional distribution of the rotavirus genotypes observed in the study population. There were statistically significant differences in the prevalence of the common genotypes identified in a region and between different regions. G9P[8] was the predominant genotype in Central Anatolia (65%) followed by G1P[8] (18%) and G3P[8] (6%), in Black Sea region (44%) followed by G1P[8] (35%) and G2P[4] (14%), in Marmara region (53%) followed by G1P[8] (25%) and G2P[8] (10%), in Mediterranean regions (49%) followed by G1P[8] (26%) and G2P[8] (13%), and in Aegean region (61%) followed by G1P[8] (19%) and G2P[8] (12%), South-East Anatolian regions (29%) followed by G1P[8] (28%) and G2P[8] (22%). In contrast, G1P[8] dominated in East Anatolia, with a rate of 51%, and G9P[8] was the second-most common genotype (31%) in the same region (Figure 4).

## Discussion

The epidemiology of rotavirus-associated diseases shows variation based on the socio-economic condition of the study population and the climate of the different countries (2, 16, 28). Although rotavirus infections can be recorded throughout the year in Turkey, the majority of rotavirus infections are observed from September to May [16]–[19], [22], [23]. In parallel to these data, we collected stool samples from infected children throughout the year and the majority of the samples (94.4%) were obtained from September to the end of May. According to previous studies in Turkey, most rotavirus infections were recorded in children aged 12 to 23 months, and a second peak was observed in the 6 to 11 month age group; more than 70% of rotavirus infections occurred in children less than 2 years of age [19], [21], [23]. These data agree with results reported from many European countries [12], [28]–[31]. Our study, conducted on high numbers of children from seven geographic regions, showed that rotavirus mainly infected children from 13 to 24 months of age followed by the 25 to 36 month age group. This current study, which provided the most comprehensive and up-to-date data, indicates that rotavirus infections in Turkey are mainly observed in children aged 13 to 36 months, who might be more prone to acquire rotavirus infection in Turkey. Therefore, further surveillance studies in Turkey should include children less than 5 years old, instead of less than 2 years old.

Although there are numerous G and P- types [4], [8], more than 85% of circulating rotavirus strains around the world are identified in five G (G1, G2, G3, G4 and G9) and three P (P[8], P[4], and P[6]) genotypes [9], [10], [32]. In agreement with these data, we found that 97.8% of rotavirus strains were in these five G genotypes and that more than 99% were in these three P types. Three common G types (G1, G2 and G9) accounted for more than 90% of the strains, and the frequency of each remaining G type was less than 5%. The predominance of common G types shows variation in different geographic locations year by year [9], [11], [15], [33]–[35]. For example, G1 was the most prevalent genotype, representing more than 70% of the rotavirus infections in North America, Europe, and Australia, but its rate was less than 30% in South America, Asia, and Africa between 1989 and 2004 [35]. A recent study carried out in Tanzania showed the predominance of G1 followed by G8, G12, and G4 [36]. In China, although G1 was the most common genotype until 2000, including more than 74% of the strains, after that time, G3 predominated [37]. In Brazil, while G1 was the most common genotype during the pre-vaccination period, after vaccination, G2 became the predominant genotype [9]. In Denmark, recent rotavirus strain surveillance from 2009 to 2013 indicates a G9/G1 replacement, and G9 has been the most predominant genotype identified among rotavirus strains in the Danish population since 2011. This surveillance study also showed an increase in the frequency of G4 and G3 genotypes [38]. In Belgium, the prevalence of G genotypes showed remarkable variation from 1999 to 2009. For example, G1 was reported as the most predominant genotype identified in more than half of the strains in 1999–2000, 2001–2002, 2005–2006, and 2007–2008. The G9 genotype dominated in 2000–2001, 2002–2003 and 2004–2005. After introduction of the rotavirus vaccines, G2 emerged as being responsible for approximately 30% of RV infections [39]. A multicenter study conducted in our neighbor country, Greece, between July 2008 and March 2010 showed that G4 (59.6%) was the most predominant genotype, followed by G1 (17.4%) during the study period [31]. Previous studies from different provinces in Turkey revealed the dominating prevalence of G1–G4 genotypes in combination with P[8] and P[4] between 2000 and 2010 [18]–[21]. From 2000 to 2002, G4 was found to be the most prevalent genotype (40.6%), followed by G1 (28.6%), G2 (8.8%), and G3(1.1%) in a study carried out on samples collected from three different regions [20]. In 2003, G1 was the most common genotype, including 75% of the strains genotyped in Izmir [23]. G1 was also the most predominant genotype (59.4%), followed by G9 (17.2%) in Ankara from 2004 to 2005 [19], shifting to G3 (38.7%) followed by G4 (25.8%) from April 2008 to February 2010 [18]. In contrast to previous studies, the current study clearly demonstrates the emergence of G9 strains, representing more than 48% of the rotavirus infections in the country from August 2012 to July 2014. These temporal and geographical variations in frequencies of rotavirus genotypes may be due to different reasons such as a selective effect of rotavirus vaccines, reassortment between the circulating strains, or the introduction of new strains [11], [33], [35], [38]. Because rotavirus vaccines have not been introduced in national vaccination programs, we can speculate that vaccine pressure is not a reason for the changes in genotype prevalence observed in our country. As concluded by Midgley et al. [38], naturally occurring selection pressures and viral evolution may explain the genotype diversity in Turkey.

In the current study, genotyping results of 1644 strains from seven geographic regions of Turkey showed that rotavirus strains distributed into high numbers (n = 42) of different G and P genotype combinations. This finding is in agreement with the results presented by Bozdayi et al. [19], who indicated 20 different types among 128 strains, and by Tapisiz et al. [17], who found 34 different genotypes among 90 strains. Although the number of different G–P combinations was very high, approximately 83.7% of rotavirus strains circulating in our study groups were defined in six common P-G combinations such as G9P[8] genotype (40.5%), followed by G1P[8] (21.6%), G2P[8] (9.3%), G2P[4] (6.5%), G3P[8] (3.5%), and G4P[8] (3.4%). These results are in agreement with the findings indicating that the five common G and P combinations (G1P[8], G2P[4], G3P[8], G4P[8], and G9P[8]) account for approximately 90% of all human rotavirus strains [12], [28], [38], [40]. We observed statistically significant differences in the prevalence of these common genotypes between different regions. G9P[8] was the most prevalent genotype in the Central Anatolia, Black Sea, Marmara, Mediterranean, South-East Anatolia, and Aegean regions. However, in East Anatolia, G1P[8] was found at high prevalence, although, the proportions of these common genotypes for each age group, gender, and month were almost similar, indicating no age, gender and seasonal bias for a particular genotype. In parallel to our data, during a five-year surveillance carried out on rotavirus-positive samples collected from eight different geographic regions in Hungary, 17 different G and P genotype combinations were reported in 2297 strains. Of these strains, 91% belonged to one of the five common genotypes (G1P[8], G2P[4], G3P[8], G4P[8], and G9P[8]), and the prevalence of these genotypes showed variations between geographic regions [12]. According to the report of EuroRotaNet, which includes genotyping results of 25,546 strains from 17 different European countries, 44 different rotavirus genotypes were identified in Europe, and G1P[8] was reported as the most prevalent genotype identified in 49.9% of the strains, followed by the G4P[8] (17%), G2P[4] (12.3%), G9P[8] (12%), and G3P[8] (5%) genotypes [28]. In contrast to our study, G1P[8] was the most predominant genotype, including 43–50% of the strains, in most European countries [12], [28], [40], [41]. However, in agreement with our results, two recent studies from Denmark [38] and Romania [42] found that G9P[8] is the most predominant genotype, with an increasing trend over time.

The genotype G9P[8] predominated from almost all participating centers in the present study, but it was the second-most common genotype observed from Erzurum province in East Anatolia. A previous study carried out on 119 children from nine provinces in four different regions of Turkey showed that G9P[8] was a less common genotype, with a rate of 3.2% in the period from 2000 to 2002 [20]. However, the increased prevalence of this genotype was observed in two studies from the Ankara province in Turkey between September 2004 and December 2005 (10%) and between January 2008 and January 2009 (19%) [17], [19] and a multicenter study from four different provinces in Turkey between October 2006 and June 2007 (25%) [43]. The importance of emerging G9P[8] is highlighted by studies in Turkey, but many study groups in different countries have also found a remarkable increase (up to 79%) in the frequency of this genotype [30], [33], [44]. G1[P8], which is used in the Rotarix vaccine, was found as the second-most prevalent genotype in the current study population. A study performed on children equal to or less than 5 years old in the Ankara province in Turkey between April 2008 and February 2010 observed G3P[8] predominance with a rate of 38.9% [18], whereas two other studies on children showed G1[P8] dominancy with the rate of 55.5% and 76% in the same province from September 2004 to June 2006 [19], [21]. In contrast to previous studies focusing on the dominance of G4P[8] (42.2%) [20] and G2[P4] (47.2%) [43], we found these genotypes at very low frequencies (3.4% and 6.5%, respectively). In Turkey, the rate of G9P[4] strains increased from 1.6% to 5.6% between September 2004 and December 2005 [19]. This rare genotype combination was found in 7.2% of the samples tested in the current study. In agreement with the idea indicating fluctuation in circulating genotypes even in the same country year by year [9], [12], [25], we observed a remarkable difference in the frequency of circulating genotypes between the results of previous studies in Turkey and those of the current study. Similar fluctuations in the prevalence of predominant genotypes were also reported from European countries [45]. For instance, in Australia, while G1P[8] (49.3%) was the predominant type followed by G2P[4] (26.1%) in 2009/2010, in 2010/2011, G2P[4] predominated (51%) followed by genotype G1P[8] (26.1%) [29], [41]. In Bulgaria, G4P[8] was reported as the most predominant genotype in 2004/2005 (56.8%) but was replaced by G9P[8] in 2005/2006 (77.7%) and by G2P[4] (41.6%) and G1P[8] (39.5%) in 2006/2007 and 2007/2008 [30]. In Hungary, while G1P[8] was reported as the predominant genotype from 2001 to 2002 (66%), its frequency decreased to 9.5% from 2005 to 2006 [46], [47]. Fluctuations in rotavirus genotype combinations observed from season to season within each country, and even from region to region within the same country, suggest the need for continuous monitoring of circulating genotypes.

Interestingly, the number of different mixed and uncommon genotypes was very high (20 different uncommon genotypes and 16 mixed types). In fact, 14.1% of the strains were in uncommon combinations and 2.2% belonged to mixed genotypes. The rate of mixed infection ranges from 2.4% to 26% in Turkey [17], [19], [20]. In parallel to our results, the rate of uncommon and mixed infections varied from 3.7% to 15% in other countries [9], [15], [48]. We found some rare combination such as G10P[8], G12P[11], G12P[6], G4P[9], G10P[4]P[8], G12P[6]P[8], G1G2P[4]P[8], G1G9P[4], G1G9P[8], and G1P[4]P[8], which have not been reported in Turkey previously [17], [19]–[21]. These data indicate that Turkey had considerably diverse rotavirus genotypes before the introduction of a nationwide vaccination program.

## Conclusion

This sentinel surveillance study carried out in 23 provinces in Turkey from August 2012 to July 2014 provides the most comprehensive and up-to-date information on rotavirus strains circulating in Turkey before the introduction of a rotavirus vaccine program. This report revealed that i) rotavirus infections mainly affect children from 13 to 24 months of age; ii) the number of different G and P genotypes was limited, whereas a very high number (42) of different G–P combinations were observed; iii) a remarkable rate of rotavirus strains were classified in uncommon and/or mixed genotypes; iv) despite heterogeneity among the genotypes observed in our study population, the G1, G2, G3, G4, and G9 genotypes included more than 97% of the rotavirus strains circulating in Turkey; v) available rotavirus vaccines have high coverage rates of rotavirus strains currently circulating in Turkey. This background information can be used to monitor the impact of rotavirus vaccines on future strain prevalence.
